# Predicting Brain Regions Related to Alzheimer's Disease Based on Global Feature

**DOI:** 10.3389/fncom.2021.659838

**Published:** 2021-05-21

**Authors:** Qi Wang, Siwei Chen, He Wang, Luzeng Chen, Yongan Sun, Guiying Yan

**Affiliations:** ^1^Academy of Mathematics and Systems Science, Chinese Academy of Sciences, Beijing, China; ^2^School of Mathematical Sciences, University of Chinese Academy of Sciences, Beijing, China; ^3^Department of Neurology, Peking University First Hospital, Beijing, China; ^4^Department of Medical Imaging, Peking University First Hospital, Beijing, China; ^5^Department of Ultrasound, Peking University First Hospital, Beijing, China

**Keywords:** Alzheimer's disease, diffusion tensor imaging, brain structural network, 2hop-connectivity, global featurescore, differential network analysis

## Abstract

Alzheimer's disease (AD) is a neurodegenerative disease that commonly affects the elderly; early diagnosis and timely treatment are very important to delay the course of the disease. In the past, most brain regions related to AD were identified based on imaging methods, and only some atrophic brain regions could be identified. In this work, the authors used mathematical models to identify the potential brain regions related to AD. In this study, 20 patients with AD and 13 healthy controls (non-AD) were recruited by the neurology outpatient department or the neurology ward of Peking University First Hospital from September 2017 to March 2019. First, diffusion tensor imaging (DTI) was used to construct the brain structural network. Next, the authors set a new local feature index 2hop-connectivity to measure the correlation between different regions. Compared with the traditional graph theory index, 2hop-connectivity exploits the higher-order information of the graph structure. And for this purpose, the authors proposed a novel algorithm called 2hopRWR to measure 2hop-connectivity. Then, a new index global feature score (GFS) based on a global feature was proposed by combing five local features, namely degree centrality, betweenness centrality, closeness centrality, the number of maximal cliques, and 2hop-connectivity, to judge which brain regions are related to AD. As a result, the top ten brain regions identified using the GFS scoring difference between the AD and the non-AD groups were associated to AD by literature verification. The results of the literature validation comparing GFS with the local features showed that GFS was superior to individual local features. Finally, the results of the canonical correlation analysis showed that the GFS was significantly correlated with the scores of the Mini-Mental State Examination (MMSE) scale and the Montreal Cognitive Assessment (MoCA) scale. Therefore, the authors believe the GFS can also be used as a new biomarker to assist in diagnosis and objective monitoring of disease progression. Besides, the method proposed in this paper can be used as a differential network analysis method for network analysis in other domains.

## Introduction

Alzheimer's disease (AD) is a neurodegenerative disease that commonly affects the elderly. It is a continuous process, from the pre-clinical stage to mild cognitive impairment (MCI) to dementia. Effective intervention in the pre-dementia or MCI stage can slow down or reverse the disease process. Therefore, early identification of patients with AD in the pre-dementia or MCI stage, as well as early and timely intervention, are of great importance to the prognosis of patients. With the development of imaging technology, the detection of AD is no longer limited to the phenomenon of abnormal protein deposition. Analysis of structural brain network information, such as brain connectome analysis, may be an effective method for early diagnosis and monitoring of disease progression (Fan et al., 2016).

Previous studies (Liu et al., 2017) have shown that changes in the topological features of the brain structural network are a hallmark of multiple neuropsychiatric disorders. Currently, there are several research efforts based on the graph theory of brain structural networks (Sanz-Arigita et al., 2010; John et al., 2017). The common method is to analyze some local features such as the degree centrality of nodes, clustering coefficient, and shortest path length of the brain structural network. Local features are difficult to be used to reveal the overall characteristics of the network. The global property by combining local properties can reveal the topological characteristics of the network more effectively, but it is never easy to choose which local indices are to be used. In this paper, the authors first defined a new local feature index, 2hop-connectivity, of the network to analyze the brain network more completely.

In this work, 20 patients with AD and 13 patients in the pre-dementia stages (non-AD) were recruited. The authors collected demographic data and clinical data and completed neuropsychological scale assessments and DTI scans. After preprocessing, the brain structural network was constructed based on the number of fibers between different brain regions. The data from the AD and the non-AD groups were analyzed to obtain the local topological features of the brain structural network. Meanwhile, the authors designed an algorithm called 2hopRWR to get the local feature index 2hop-connectivity, and then a new index global feature score (GFS) was proposed by combing four classical local features and 2hop-connectivity. As a result, the authors predicted and analyzed the top 10 brain regions based on the difference of the GFS scores between the AD and the non-AD groups. Then, the authors analyzed the correlation between the GFS and the cognitive scale scores by performing canonical correlation analysis (CCA). Finally, the strengths and limitations of the work presented in this paper and its prospects are discussed.

## Materials and Methods

### Data Collection and Pre-Processing

#### Research Participants

A case–control study design was adopted in this study. Patients with AD who were admitted to the neurology outpatient department or the neurology ward of Peking University First Hospital from September 2017 to March 2019 were recruited in the study. Normal controls were recruited at the same time. The inclusion criteria for the AD group were as follows: (1) Han nationality, over 18 years old, who were right-handed and agreed to participate in this study; (2) patients diagnosed as probable AD clinically according to the 2011 National Institute on Aging and the Alzheimer's Disease Society (NIA-AA) diagnostic criteria (McKhann et al., 2011); (3) no serious white matter lesions found by MRI examination, which meant that the Fazekas scale score was no more than 2. The inclusion criteria for the normal control group were as follows: (1) Han nationality, over 18 years old, who were right-handed and agreed to participate in this study, so as to match their ages with the ages of subjects of the AD group; (2) normal cognitive function and not meeting the diagnostic criteria of dementia; (3) no serious white matter lesions found on MRI examination, which meant that the Fazekas scale score was no more than 2. The exclusion criteria were as follows: (1) patients or their family members refusing to participate in the study; (2) unable to complete 3.0Tesla MRI examination due to various reasons; (3) with a history of cerebrovascular diseases, or cognitive impairment caused by toxication, metabolic disease, infection, autoimmune disease, or drug and with a history of demyelination of the central nervous system, white matter lesions, or other diseases that may affect the white matter structure of the brain; (4) with a history of serious mental illness, such as depression, mania, and schizophrenia; (5) having a long-term history of alcoholism or vegetarianism; (6) Patients with other types of dementia other than AD.

This study was approved by the Clinical Research Ethics Committee of Peking University First Hospital. All participants or their family members signed the informed consent.

#### Clinic Data Collection

In this research, 20 patients with AD and 13 healthy controls (non-AD) were recruited. Demographic information and the medical history of the participants were collected. Blood tests were conducted to exclude cognition impairment caused by other reasons. All the patients were assessed on a set of neuropsychological scales by the same trained neurologist to assess overall cognitive function including memory, executive function, language, and visuospatial and structural abilities of all the participants. The ability of daily living and mental and behavioral symptoms of the subjects were also evaluated (Supplementary Table 1).

#### Brain Imaging Examination

Brain imaging examinations were performed by the Department of Imaging, Peking University First Hospital. The participants received imaging examination within 1 week before or after the completion of the scales. The images were collected by using a GE Discovery MR750 3.0Tesla MRI scanner. The acquisition sequence included the following: axial T1-weighted imaging, axial T2-weighted imaging, and high-resolution DTI sequences. Some of the sequence parameters were as follows:

Axial T1-weighted imaging: Repetition time (TR) = 2,500 ms. Echo time (TE) = 24.0 ms. Field of vision (FOV) = 24.0 cm × 24.0 cm. Layer thickness (ST) = 5 mm.

Axial T2-weighted imaging: TR = 8,400 ms. TE = 140.0 ms. FOV = 20.0 cm × 20.0 cm. ST = 3 mm.

DTI: TR = 4,600 ms. TE = 90.0 ms. FOV = 24.0 cm × 24.0 cm. ST = 4 mm. The dispersion sensitive gradient was applied in 25 directions, and 36 layers of images were scanned in each direction, b = 1,000 s/mm^2^. Besides, there was a group of images without dispersion weighting, b = 0.

#### DTI Data Processing and Brain Network Construction

In this study, PANDA (Pipeline for Analyzing braiN Diffusion imAges) (Cui et al., 2013), a toolkit based on MATLAB (R2009b; MathWorks) and FSL that integrates several processing steps, was used to process the data and construct the network. After preprocessing, which included the import of the DICOM format file, scalp and skull removing, brain tissue cutting, eddy current effect correction and head movement correction, multiple diffusion-weighted image acquisition, and processing could be finished.

The images of all individuals were placed in the standardized template and the eigenvalues of each dispersion were measured. The FA (fractional anisotropy) image of each individual was non-linearly loaded into the FA template (FMRIB58_FA_TEMPLATE) of the Montreal Neurological Institute (MNI) space. Through the results of transformation, the diffuse eigenvalues of each individual in the MNI space were resampled and the space was partitioned (the resolutions were 1 mm × 1 mm × 1 mm and 2 mm × 2 mm × 2 mm).

The deterministic fiber-tracking method, FACT [fiber assignment by continuous tracking [Mori and van Zijl, 2002]], was then used to construct the brain structural network. The FA threshold was set to be 0.2–1 and the angle threshold was set to be 45°, that is, when the FA was <0.2 or the tracking angle was >45°, the fiber tracking would end. The ICBM152 AAL−90 [Automated Anatomical Labeling, AAL [Tzourio-Mazoyer et al., 2002)] brain atlas was used to divide the brain of each subject into 45 left and right symmetrical brain regions, with 90 brain regions in total. Each node represented a brain region in the brain structural network. The fiber connection between any two brain regions was represented by an edge, and the edge weight represented the fiber number (FN). The FN matrix of 90 brain regions was obtained by using PANDA (Cui et al., 2013).

Mathematically, the authors regarded 90 brain regions and their fiber connection as a weighted graph *G*(*V, E, W*), and *V* = {*v*_1_, *v*_2_, …, *v*_90_}, *E* = {*e*_*v*_*i*_*v*_*j*__, *v*_*i*_ ≠ *v*_*j*_}, *W* = {*w*_*v*_*i*_*v*_*j*__}, where *v*_*i*_ denotes the *i-*th brain region, *e*_*v*_*i*_*v*_*j*__ is the edge if there were fiber connection between brain region *v*_*i*_ and *v*_*j*_, and *w*_*v*_*i*_*v*_*j*__ is the edge weight that is the fiber number between the brain region *v*_*i*_ and *v*_*j*_. The average value of the FN matrix of AD was calculated by adding the FN matrix of each patient with AD and dividing it by the number of patients with AD. Similarly, the authors took the average value of the FN matrix of all normal controls to the FN matrix of the non-AD group.

### Local Features

#### Degree Centrality

Let *d*(*v*_*i*_) denote the degree of a node *v*_*i*_, which is the number of nodes associated with *v*_*i*_. And the degree centrality of a node *v*_*i*_ is defined as follows:

(1) $$\begin{array}{l} C_{D}\left(v_{i}\right)=\frac{d\left(v_{i}\right)}{n-1} \end{array}$$

#### Betweenness Centrality

Betweenness centrality *c*_*B*_ of a node *v*_*i*_ is the sum of the fraction of the shortest paths of all pairs that pass through *v*_*i*_:

(2) $$\begin{array}{l} c_{B}\left(v_{i}\right)=\underset{v_{s},v_{t}\in V}{\sum }\frac{\sigma \left(v_{s},v_{t}|v_{i}\right)}{\sigma \left(v_{s},v_{t}\right)}, \end{array}$$

where σ(*v*_*s*_, *v*_*t*_) is the number of the shortest paths between *v*_*s*_ and *v*_*t*_, and σ(*v*_*s*_, *v*_*t*_|*v*_*i*_) is the number of the shortest paths passing through the node *v*_*i*_. If *s* = *t*, σ(*v*_*s*_, *v*_*t*_) = 1, and if *i* = *s* or *i* = *t*, σ(*v*_*s*_, *v*_*t*_|*v*_*i*_) = 0.

In short, if the shortest path between many nodes in the network passes through a point *v*, then *v* has a high degree of betweenness centrality. This node is on the shortcut between other node pairs.

#### Closeness Centrality

Closeness centrality *C*_*c*_ of a node *v*_*i*_ is the reciprocal of the sum of the shortest path distances from *v*_*i*_ to all *n* − 1 other nodes. Since the sum of distances depends on the number of nodes in the graph, closeness is normalized by *n* − 1.

(3) $$\begin{array}{l} C_{c}\left(v_{i}\right)=\left(n-1\right)/\overset{n}{\underset{\begin{array}{c} j=1\\ j\neq i \end{array}}{\sum }}d\left(v_{j},v_{i}\right), \end{array}$$

where *d*(*v*_*j*_, *v*_*i*_) is the shortest path distance between *v*_*j*_ and *v*_*i*_, and *n* is the number of nodes in the graph.

Closeness centrality is the sum of the distance from a node to all other nodes. The smaller the sum, the shorter the path from this node to all other nodes is, and the closer the node is to all other nodes. It reflects the proximity between a node and other nodes.

#### Number of Maximal Cliques

In graph theory, the clique of graph *G* is a complete subgraph *H* of *G*. *H* is a maximal clique of graph *G* if it is not included by any other clique. The number of maximal cliques of a node can reflect the closeness between the node and other nodes. Only when multiple nodes are all connected can they be considered as maximal cliques. In this paper, the authors use *N*_*MC*_(*v*_*i*_) to represent the number of maximal cliques for node *v*_*i*_.

#### 2hop-Connectivity

When examining the correlation between any two nodes in the network, most network analysis methods only consider whether there is an edge connection between two nodes, i.e., if there is an edge, the correlation is high, and if there is no connection, the correlation is weak. In this case, if the edges of the graph are missing due to the disturbance, the results may be highly biased. For example, for the general random walk (RW) algorithm, the state transition probability is determined by the adjacency matrix of the network. If the adjacency matrix is disturbed, its steady-state probability will change. Generally, when analyzing the correlation between network nodes, the correlation between unconnected nodes in the network will be very low, which makes it difficult to discover the potential characteristics of the network. For any two different nodes in the network, in order to describe the correlation more accurately, this work considers not only the first-order neighbors between the nodes but also the second-order neighbors between the nodes, and an algorithm called 2hopRWR is proposed. Finally, each node can get a novel local feature index 2hop-connectivity, whose numerical magnitude is represented by the local feature score *S*_2−*hop*_. The importance of a node can be judged based on *S*_2−*hop*_; the larger the *S*_2−*hop*_, the more important the node is.

##### 2-Hop Random Walk With Restart Algorithm

The general random walk on the graph is a transition process that involves moving from a given node to a randomly selected neighboring node at each step. Therefore, the set of nodes {*v*_1_, *v*_2_, …, *v*_*n*_} is considered as a set of states {*s*_1_, *s*_2_, …, *s*_*n*_} in a finite Markov chain M. The transition probability of M is a conditional probability defined as *P*(*v*_*j*_, *v*_*i*_) = *Prob*(*s*_*t*+1_ = *v*_*i*_ | *s*_*t*_ = *v*_*j*_), which implies that the M will be at *v*_*i*_ at time *t* + 1 given that it was at *v*_*j*_ at time *t*. M is homogeneous because the transition probability from one state to another is independent of time *t*. Moreover, for any *v*_*j*_ of *V*, there is $\underset{v_{i}\in V}{\sum }P\left(v_{j},v_{i}\right)$ = 1. Note that M is memoryless, so the transition matrix *P* ∈ ℝ^|*V*|×|*V*|^ of M can be defined.

In general, the transition probability *P*(*v*_*j*_, *v*_*i*_) is defined as follows:

(4) $$\begin{array}{l} P\left(v_{j},v_{i}\right)=\frac{1}{d\left(v_{j}\right)}. \end{array}$$

Denote *D*_*G*_ = *diag*{*d*_1_, *d*_2_, …, *d*_*n*_} as the diagonal matrix, where $d_{i}=\overset{n}{\underset{j=1}{\sum }}w_{v_{i}v_{j}}$. Thus, *P* can be rewritten in a matrix notation, given as follows:

(5) $$\begin{array}{l} P=D_{G}^{-1}W. \end{array}$$

Define $r_{t}\in {\mathbb{R}}^{|V|\times 1}$ as a vector in which the *i*-th element represents the probability of discovering the random walk at node *v*_*i*_ at step *t*, so the probability *r*_*t*+1_ can be calculated iteratively by:

(6) $$\begin{array}{l} r_{t+1}=P^{T}r_{t}. \end{array}$$

For the random walk with restart (RWR) algorithm (Tong et al., 2006–2006), there is an additional restart item compared to the above algorithm. The probability *r*_*t*+1_ can be calculated iteratively by using the following expression:

(7) $$\begin{array}{l} r_{t+1}=cP^{T}r_{t}+\left(1-c\right)r_{0}. \end{array}$$

Define initial probability $r_{0}\in {\mathbb{R}}^{|V|\times 1}$ as a vector in which the *i*-th element is equal to 1, while other elements are 0s. And 1 − *c* is the restart probability (0 ≤ *c* ≤ 1).

**Figure 1:**
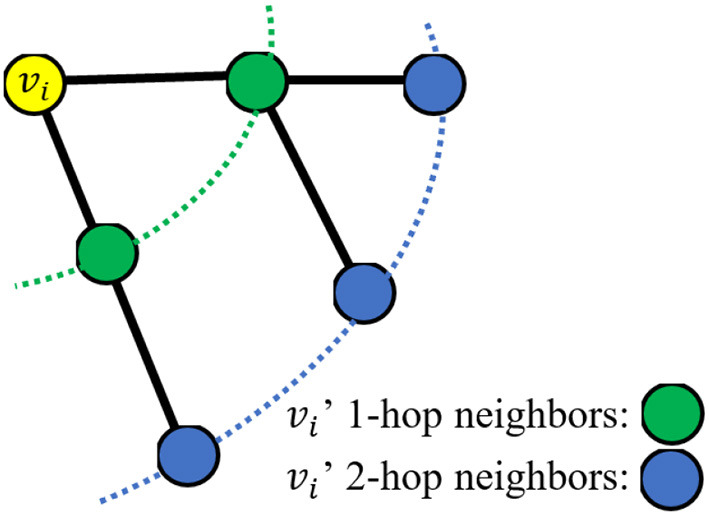
A schematic diagram of 1-hop and 2-hop neighbors.

However, the RW and RWR algorithms are based on the 1-hop neighbor relations, which means that the random walk is based on the existing edges of the graph. Figure 1 is the schematic diagram of 1-hop and 2-hop. If some edges of the graph are missing, the corresponding nodes cannot be directly transferred, which will lead to a large deviation in the steady-state probability. Therefore, the effectiveness of these algorithms is too dependent on the integrity of the graph structure. So in this work, in addition to the 1-hop neighbor relations, the 2-hop neighbors are also considered and a novel RW algorithm called 2hopRWR is proposed.

The probability *r*_*t*+1_ can be calculated iteratively by:

(8) $$\begin{array}{l} r_{t+1}=c\left(\alpha_{1}P^{T}+\alpha_{2}{\left(P^{2}\right)}^{T}\right)r_{t}+\left(1-c\right)r_{0}, \end{array}$$

where α_1_ and α_2_ are the percentages of choosing 1-hop and 2-hop neighbors, respectively. Specifically, for each point *v*_*i*_ ∈ *V*, α_1_ is the ratio of the number of 1-hop neighbors to the total number of 1-hop and 2-hop neighbors, α_2_ is the ratio of the number of 2-hop neighbors to the total number of 1-hop and 2-hop neighbors. Therefore, α_1_ + α_2_ = 1.

At the beginning of the 2hopRWR, a starting node *v*_*i*_ is chosen; then, it would have a probability of *c* to walk to other nodes and have a probability of 1 − *c* to stay in place. Specifically, when the process of walk reaches the node *v*_*j*_, it has a probability of α_1_*c* to walk based on existing edges to 1-hop neighbors and has a probability of α_2_*c* to walk to 2-hop neighbors, and it also has a probability of 1 − *c* to restart the walk, i.e., to go back to the node *v*_*i*_.

**Figure 2:**
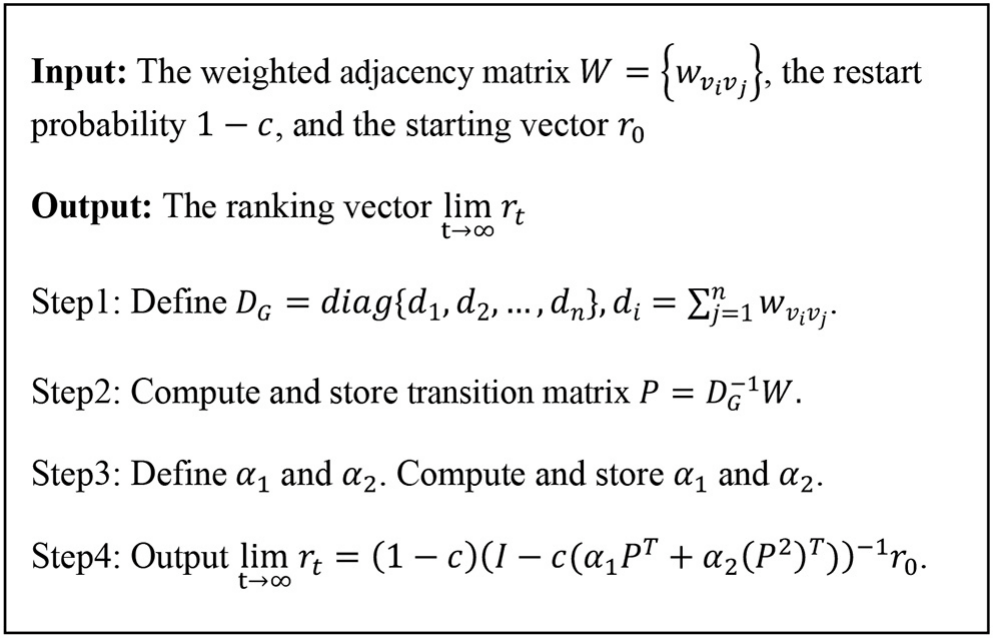
2hopRWR algorithm framework.

After some steps, the 2hopRWR will be stable, i.e., when *t* tends to infinity, *r*_*t*+1_ = *r*_*t*_. The proof is given in Section Proof of Convergence. When the 2hopRWR is stable, steady-state probability between node *v*_*i*_ and node *v*_*j*_ is defined as the *j*-th element of *r*_*t*_ corresponding to the starting node is *v*_*i*_. Figure 2 is the flowchart of 2hopRWR algorithm.

##### Proof of Convergence

Here, the authors will prove that the RWR algorithm is convergent, i.e., for Equation (8) when *t* tends to infinity, *r*_*t*+1_ = *r*_*t*_.

Define:

(9) $$\begin{array}{l} M=c\left(\alpha_{1}P^{T}+\alpha_{2}{\left(P^{2}\right)}^{T}\right), \end{array}$$

(10) $$\begin{array}{l} N=\left(1-c\right){\left(I-c\left(\alpha_{1}P^{T}+\alpha_{2}{\left(P^{2}\right)}^{T}\right)\right)}^{-1}. \end{array}$$

Thus, using (9) and (10) we get:

(11) $$\begin{array}{l} r_{t+1}-Nr_{0}=M\left(r_{t}-Nr_{0}\right). \end{array}$$

Define,

(12) $$\begin{array}{l} B_{t}=r_{t}-Nr_{0}. \end{array}$$

Then,

(13) $$\begin{array}{l} B_{t+1}=MB_{t}. \end{array}$$

By (13), when t = 0, we have *B*_0_ = (*I* − *N*)*r*_0_, thus:

(14) $$\begin{array}{l} B_{t}=M^{t}\left(I-N\right)r_{0}, \end{array}$$

(15) $$\begin{array}{l} r_{t}=\left[N+M^{t}\left(I-N\right)\right]r_{0}. \end{array}$$

Since $\underset{\text{t}\to \infty }{\lim}M^{t}=0$, we have:

(16) $$\begin{array}{l} \underset{\text{t}\to \infty }{\lim}r_{t}=Nr_{0}=\left(1-c\right){\left(I-c\left(\alpha_{1}P^{T}+\alpha_{2}{\left(P^{2}\right)}^{T}\right)\right)}^{-1}r_{0}. \end{array}$$

Hence,

(17) $$\begin{array}{l} \underset{\text{t}\to \infty }{\lim}r_{t+1}-r_{t}=0, \end{array}$$

which implies that the convergence of the algorithm is proved.

##### Comparison With Other Second-Order Methods

**Figure 3:**
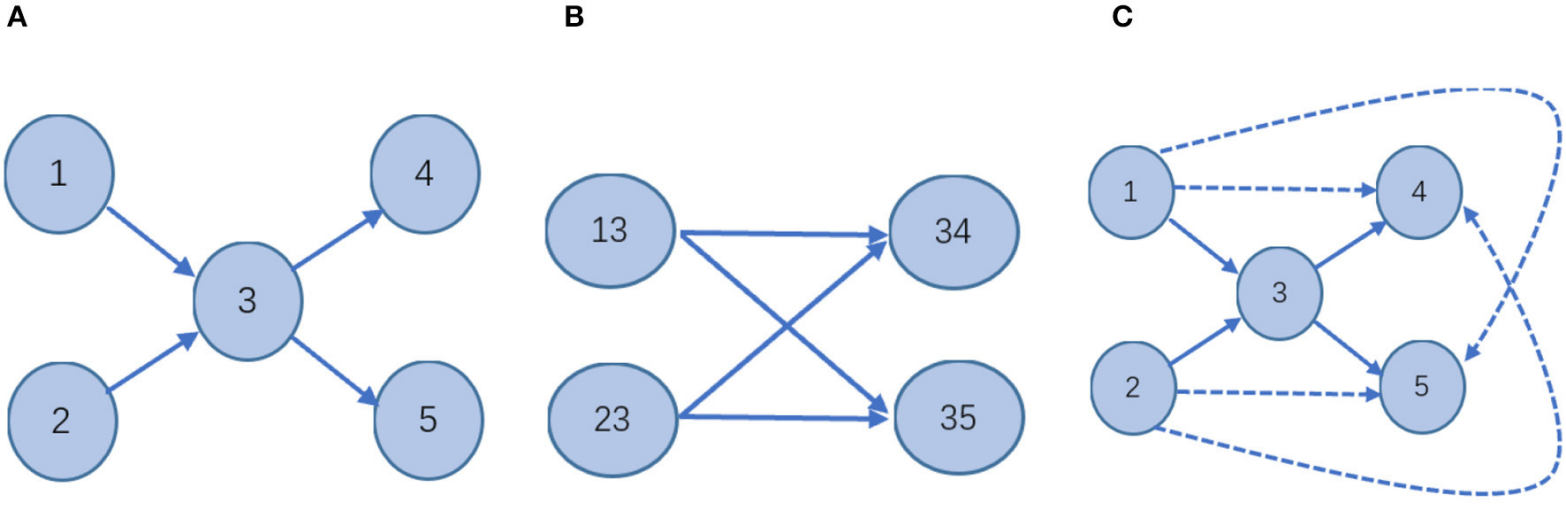
Schematic—different stochastic processes on the network. **(A)** In a first-order Markov model, the state space is isomorphic to the physical network: every node corresponds to one state; every link indicates a transition between those states. It is a Markov stochastic process. **(B)** In the second-order Markovian model, the state space is different from **(A)**. In this case, the probability of moving from one node to another will appear non-Markovian. It is a non-Markovian stochastic process. **(C)** 2hopRWR is a first-order Markov model. A solid line indicates that the state is reachable in one step between states. The dashed lines indicate states that are reachable in one step to the second-order states with some probability. It is a Markov stochastic process.

Considering higher-order structural properties of a network is not a new idea. Certain works in the literature (Salnikov et al., 2016; Benson et al., 2017) present the spacey random walk, a non-Markovian stochastic process whose stationary distribution is given by the tensor eigenvector. The higher-order structural properties of a network of Salnikov et al. (2016) and Benson et al. (2017) mean the transitions depend on the past few states, rather than just the last one. The process is called a higher-order Markov chain which is not a Markov chain. For example, for a directed graph network (Figure 3), the two definitions of higher-order information are compared.

Therefore, it is clear that the higher-order information on the network defined in the literature (Salnikov et al., 2016; Benson et al., 2017) is obtained by a random walk of the non-Markovian process, while the 2hopRWR is obtained by a random walk of the Markovian process. For the case of missing data, i.e., when the links between the nodes are missing, the second-order states [e.g., (13)] in Figure 3B constructed by the method of literature (Salnikov et al., 2016; Benson et al., 2017) will have a large error impact, while 2hopRWR can reduce this part of the impact because 2hopRWR attenuates the weight of the first-order connections.

##### 2Hop-Connectivity Feature Score

Any two nodes can be ranked twice according to 2hopRWR. Define Π = {π_*ij*_}_|*V*|×|*V*|_ as the steady-state probability matrix, where π_*ij*_ indicates the steady-state probability between node *v*_*i*_ and node *v*_*j*_, i.e., the probability of 2hopRWR starts from node *v*_*i*_ and reaches node *v*_*j*_ when the process is stable. In short, the value of π_*ij*_ is the *j*-th element of steady-state probability $\underset{\text{t}\to \infty }{\lim}r_{t}$ when the *i*-th element of *r*_0_ is 1. Therefore, the local feature score *S*_2−*hop*_ for node *v*_*i*_ is defined as follows:

(18) $$\begin{array}{l} S_{2-hop}\left(v_{i}\right)=\overset{\left|V\right|}{\underset{j=1}{\sum }}\pi_{ij}. \end{array}$$

The 2hop-connectivity exploits the higher-order information of the graph structure relative to the traditional graph theory metrics. Since there is a certain amount of noise in DTI data, a certain threshold is chosen for denoising when converting DTI data into brain networks, i.e., when the number of fibers between two brain regions is low (lower than the threshold), no fiber tracts (edges) are considered between two brain regions (nodes). However, since there are inherently fewer fiber tract connections between brain regions in patients with AD, the brain network constructed after denoising may differ significantly from the true situation. The 2hop-connectivity is insensitive to 1-hop order relations and more robust to changes in the network structure, so 2hop-connectivity is instead more effective for sparse networks (e.g., structural brain networks in patients with AD).

### Global Feature

In this paper, the authors consider the integration of local features to obtain a new network index: global feature.

The authors normalized the component of different feature scores to [0, 1]. The normalized features are recorded as *NC*_*D*_, *NC*_*B*_, *NC*_*C*_, *NN*_*MC*_, and *NS*_2−*hop*_.

Then, define global feature score *GFS* for node *v*_*i*_ as follows:

(19) $$\begin{array}{l} GFS\left(v_{i}\right)=\alpha_{1}NC_{D}\left(v_{i}\right)+\alpha_{2}NC_{B}\left(v_{i}\right)+\alpha_{3}NC_{C}\left(v_{i}\right)\\ \;+\alpha_{4}NN_{MC}\left(v_{i}\right)+\alpha_{5}NS_{2-hop}\left(v_{i}\right) \end{array}$$

**Figure 4:**
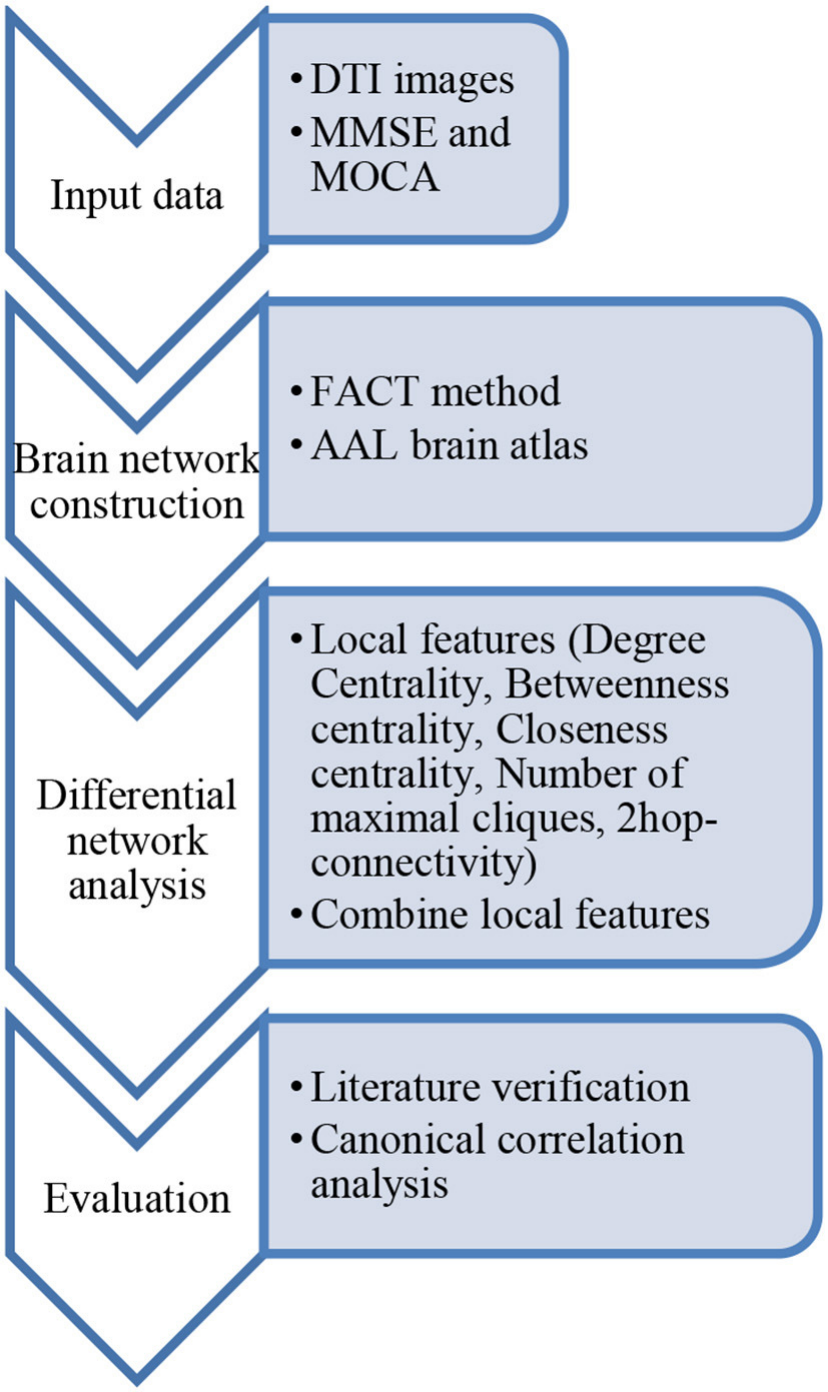
Workflow of the approach presented in this paper.

In general, let the weight α_*i*_ = 1/5 (*i* = 1, 2, 3, 4, 5), and it also can be addressed by regression over AD-group's Mini Mental State Examination (MMSE) data. For example, using the MMSE scale or Montreal Cognitive Assessment (MoCA) scale data in Supplementary Table 1, the correlation between the five characteristic scores of the AD group with the MMSE or MoCA scale was analyzed. If the *GFS* of a node is relatively high, it means that the node plays a key role in the network. Figure 4 is the flowchart of our method.

## Results

### Top Ten Brain Regions for Literature Verification

By comparing the GFS of the non-AD group with that of the AD group, the authors got the top ten brain regions in GFS scoring difference (i.e., *GFS*_*non*−*AD*_ (*v*_*i*_) − *GFS*_*AD*_ (*v*_*i*_)). Then, literature verification was carried out to find out whether these brain regions were related to AD, and the results are shown in Table 1.

**Table 1:** Top 10 brain regions in GFS scoring difference between AD and non-AD groups.

**Rank**	**Region ID**	**AAL regions**	**Evidence**
1	40	ParaHippocampal_R	van Hoesen et al., 2000
2	3	Frontal_Sup_L	Perri et al., 2005
3	37	Hippocampus_L	Du et al., 2001
4	42	Amygdala_R	Tsuchiya and Kosaka, 1990
5	22	Olfactory_R	Wilson et al., 2009
6	78	Thalamus_R	Ryan et al., 2013
7	15	Frontal_Inf_Orb_L	Liu et al., 2020
8	9	Frontal_Mid_Orb_L	Li et al., 2019
9	68	Precuneus_R	Karas et al., 2007
10	38	Hippocampus_R	Du et al., 2001

**Figure 5:**
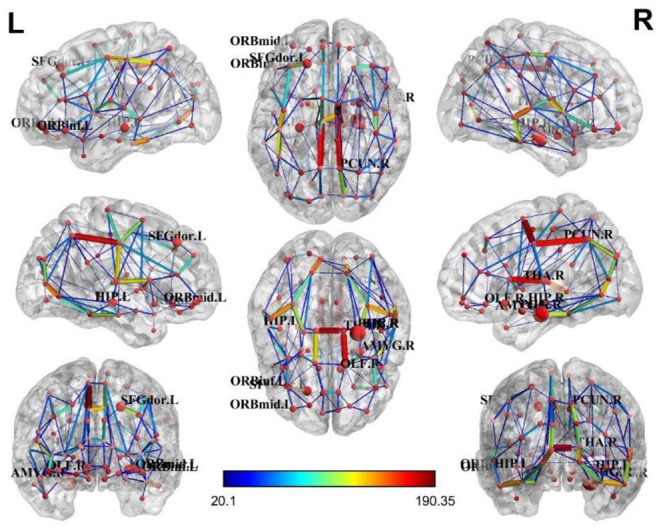
Brain structural network of the AD group.

**Figure 6:**
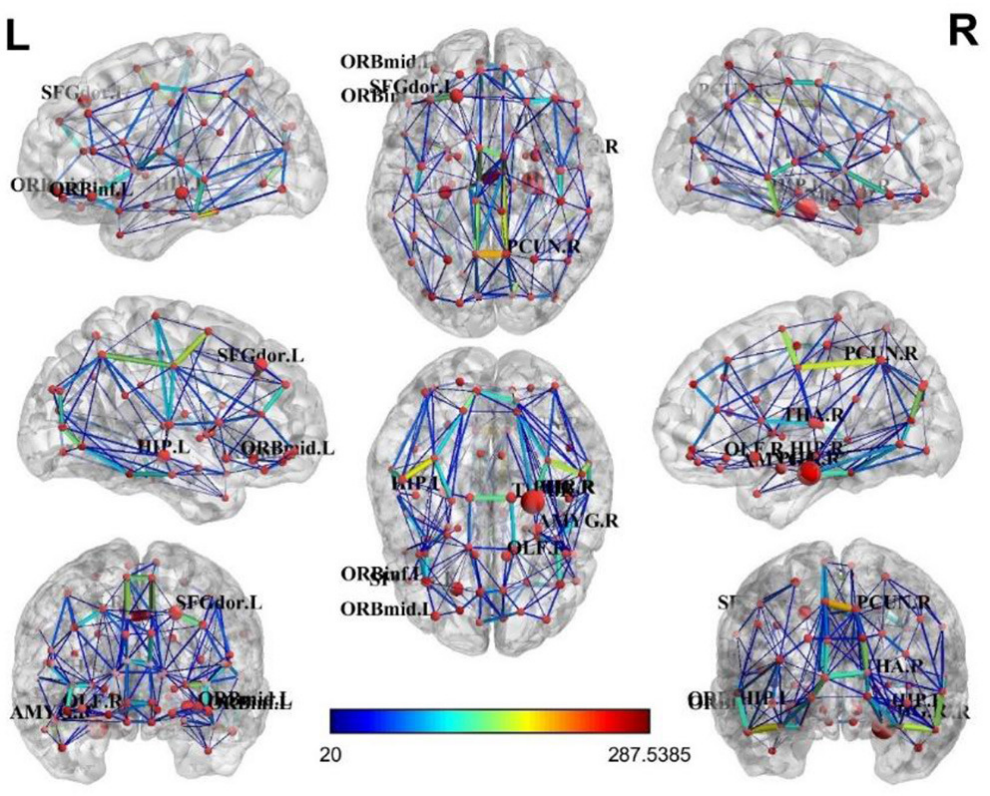
Brain structural network of the non-AD group.

For the brain structure network of the AD and non-AD groups, visualization results (Manning et al., 2014) showed a relative concentration of the top ten brain regions, as shown in Figures 5, 6.

### Compare GFS With Local Features

More importantly, the authors compared the respective predictive effects of all local features. As can be learned from Table 2, whether it is the top 10, 20, 30, or 40% brain regions with the largest differences, the predictive effect (Table 2) of GFS is always better than the other local features from the results validated in the literature, as detailed in the Supplementary Data Sheet 1.

**Table 2:** Comparison of the proportion of verified AD-related brain regions for top 10, 20, 30, and 40% ranked by different measures.

**(%)**	**NC_D (%)**	**NC_B (%)**	**NC_C (%)**	**NN_MC (%)**	**NS_2-hop (%)**	**GFS (%)**
Top 10	**100.00**	**100.00**	**100.00**	**100.00**	**100.00**	**100.00**
Top 20	88.89	94.44	94.44	83.33	83.33	**100.00**
Top 30	85.19	92.59	88.89	85.19	70.37	**96.30**
Top 40	80.56	80.56	**86.11**	80.56	75.00	**86.11**

*The bold values indicate the best results*.

### Canonical Correlation Analysis

In this section, the authors analyze whether GFS in the top ten brain regions correlates with the results of MMSE and MoCA. It allows for the analysis of whether there is a correlation between two sets of variables. Since some people were illiterate, so they could not complete the MoCA test; the authors took all the people who completed two scales, which were 29 in total (19 AD and 10 non-AD). At this point, canonical correlation analysis can be used. The basic principle is that to grasp the correlation between the two groups of variables as a whole, two composite variables U and V (linear combination of the two groups of variables) are extracted from the two groups of variables, respectively, and the correlation between the two composite variables is used to reflect the overall correlation between the two groups of variables.

The results of the canonical correlation analysis showed that the correlation coefficient between the typical variable pair 1 is 0.7136, which means that there is a very strong correlation between GFS and MMSE/MoCA scale information.

## Discussion

In this paper, a new local feature index 2hop-connectivity was set up to measure the correlation between different regions. 2hop-connectivity is a node importance metric, just like graph-theoretic metrics such as degree centrality. Since most biological networks are relatively sparse, 2hop-connectivity may work better for node importance metrics of sparse networks. And for this purpose, a novel random walk algorithm 2hopRWR is proposed, which can calculate the local feature index 2hop-connectivity. Also, the proof of convergence is given. Next, the idea of combining five local features to obtain the GFS was used in this paper, which is more convincing than using a single network parameter to describe the importance of network nodes. Then, the results of literature verification and canonical correlation analysis also verify the rationality and the validity of the proposed method. Thus, GFS can be used to distinguish DTI images of the AD and the non-AD groups. Finally, the top 10 brain regions in the GFS scoring difference predicted in this paper have been validated in the literature. Literature validation results comparing GFS with local features showed that GFS outperforms individual local features. However, the brain network constructed in this paper is a structural network, and the structure and function should be combined in the future. For example, more results may be obtained upon combining the available fMRI data and analyzing the differences of different brain regions in different tasks. For the importance evaluation of network nodes, there is no perfect standard measurement at present, and it is difficult to evaluate the advantages and disadvantages of the node importance algorithm. Another important problem is the need to design evaluation indicators to measure the importance of nodes.

In brief, GFS is expected to be an important and useful index for identifying the difference between network nodes and detecting the changes in information transmission between brain regions in patients with AD. Moreover, it may provide useful insights into the underlying mechanisms of AD. Many elder test subjects are illiterate and are not able to perform the MMSE and MoCA scales properly, so the GFS can be used as a diagnostic aid to infer whether a subject may be a patient with AD from DTI imaging data alone, which can greatly reduce the workload of medical practitioners. Finally, GFS can be used as a differential network analysis method (Lichtblau et al., 2017) in other areas of network analysis. The authors also expect the application of the 2hopRWR algorithm in traditional network analysis tasks, such as node classification, link prediction, and graph classification.

## Data Availability Statement

The raw data supporting the conclusions of this article will be made available by the authors, without undue reservation.

## Author Contributions

QW designed the work, developed the computing method, wrote the code, analyzed the result, and wrote the manuscript. SC collected clinical data, analyzed the statistics, and polished the manuscript. HW and LC contributed to the imaging work and the statistical analysis. YS designed the work, collected clinical data, and contributed with theoretical support. GY designed the work, analyzed the result, and revised the manuscript. All authors contributed to the article and approved the submitted version.

## Conflict of Interest

The authors declare that the research was conducted in the absence of any commercial or financial relationships that could be construed as a potential conflict of interest.

## Supplementary Material

Supplementary Table 1 MMSE & MoCA data.

Supplementary Table 2 DTI data overview.

Supplementary Data Sheet 1 Results of literature validation for GFS and local features.
