# Supplementary material for: Predicting Brain Regions Related to Alzheimer's Disease Based on Global Feature
Source: Front Comput Neurosci. 2021 May 21;15:659838. doi: 10.3389/fncom.2021.659838 (PMC8175859; doi:10.3389/fncom.2021.659838)
Supplement: Supplementary Data Sheet 1 — Results of literature validation for GFS and local features. [file Data_Sheet_1.PDF]

SUPPLEMENTARY TABLE 1 | Literature validation results of brain regions related to Alzheimer's Disease (AD) in AAL template (90 regions). In particular, the relevant kinds of the literature of region ID 77, 78, 81, 82, 85, and 86 were found based on literature (Zhang et al., 2015)

Region ID	AAL regions	Abbreviation	Evidence
1	Precental gyrus	PreCG.L	(Kang et al., 2013b)
2	Precental gyrus	PreCG.R	No literature was found
3	Superior frontal gyrus, dorsolateral	SFGdor.L	(Qin et al., 2019)
4	Superior frontal gyrus, dorsolateral	SFGdor.R	No literature was found
5	Superior frontal gyrus, orbital part	ORBsup.L	No literature was found
6	Superior frontal gyrus, orbital part	ORBsup.R	No literature was found
7	Middle frontal gyrus	MFG.L	(Schultz et al., 2015)
8	Middle frontal gyrus	MFG.R	(Schultz et al., 2015)
9	Middle frontal gyrus, orbital part	ORBmid.L	(Li et al., 2019)
10	Middle frontal gyrus, orbital part	ORBmid.R	(Li et al., 2019)
11	Inferior frontal gyrus, opercular part	IFGoperc.L	No literature was found
12	Inferior frontal gyrus, opercular part	IFGoperc.R	No literature was found
13	Inferior frontal gyrus, triangular part	IFGtriang.L	No literature was found
14	Inferior frontal gyrus, triangular part	IFGtriang.R	No literature was found
15	Inferior frontal gyrus, orbital part	ORBinf.L	(Liu et al., 2020)
16	Inferior frontal gyrus, orbital part	ORBinf.R	(Shan et al., 2018)
17	Rolandic operculum	ROL.L	No literature was found
18	Rolandic operculum	ROL.R	No literature was found
19	Supplementary motor area	SMA.L	No literature was found
20	Supplementary motor area	SMA.R	No literature was found
21	Olfactory cortex	OLF.L	(Reyes et al., 1986)
22	Olfactory cortex	OLF.R	(Reyes et al., 1986)
23	Superior frontal gyrus, medial	SFGmed.L	(Hallam et al., 2020)
24	Superior frontal gyrus, medial	SFGmed.R	(Hallam et al., 2020)
25	Superior frontal gyrus, medial orbital	ORBsupmed.L	(Hallam et al., 2020)
26	Superior frontal gyrus, medial orbital	ORBsupmed.R	(Hallam et al., 2020)
27	Gyrus rectus	REC.L	(Mölsä et al., 1987)
28	Gyrus rectus	REC.R	(Mölsä et al., 1987)
29	Insula	INS.L	(Foundas et al., 1997)
30	Insula	INS.R	(Foundas et al., 1997)
31	Anterior cingulate and paracingulate gyri	ACG.L	No literature was found
32	Anterior cingulate and paracingulate gyri	ACG.R	No literature was found
33	Median cingulate and paracingulate gyri	DCG.L	No literature was found
34	Median cingulate and paracingulate gyri	DCG.R	No literature was found
35	Posterior cingulate gyrus	PCG.L	(Scheff et al., 2015)
36	Posterior cingulate gyrus	PCG.R	(Scheff et al., 2015)
37	Hippocampus	HIP.L	(Foundas et al., 1997)
38	Hippocampus	HIP.R	(Foundas et al., 1997)

39	Parahippocampal gyrus	PHG.L	(Eskildsen et al., 2015)
40	Parahippocampal gyrus	PHG.R	(Eskildsen et al., 2015)
41	Amygdala	AMYG.L	(Tsuchiya and Kosaka, 1990)
42	Amygdala	AMYG.R	(Tsuchiya and Kosaka, 1990)
43	Calcarine fissure and surrounding cortex	CAL.L	(Ren et al., 2020)
44	Calcarine fissure and surrounding cortex	CAL.R	(Ren et al., 2020)
45	Cuneus	CUN.L	No literature was found
46	Cuneus	CUN.R	No literature was found
47	Lingual gyrus	LING.L	(Lehmann et al., 2013)
48	Lingual gyrus	LING.R	(Lehmann et al., 2013)
49	Superior occipital gyrus	SOG.L	(Beyer et al., 2012)
50	Superior occipital gyrus	SOG.R	(Beyer et al., 2012)
51	Middle occipital gyrus	MOG.L	(Lehmann et al., 2013)
52	Middle occipital gyrus	MOG.R	(Lehmann et al., 2013)
53	Inferior occipital gyrus	IOG.L	(Liu et al., 2018)
54	Inferior occipital gyrus	IOG.R	No literature was found
55	Fusiform gyrus	FFG.L	(Ma et al., 2020)
56	Fusiform gyrus	FFG.R	(Ma et al., 2020)
57	Postcentral gyrus	PoCG.L	(Kang et al., 2013b)
58	Postcentral gyrus	PoCG.R	(Kang et al., 2013b)
59	Superior parietal gyrus	SPG.L	(Vasconcelos et al., 2014)
60	Superior parietal gyrus	SPG.R	(Vasconcelos et al., 2014)
61	Inferior parietal, but supramarginal and angular gyri	IPL.L	No literature was found
62	Inferior parietal, but supramarginal and angular gyri	IPL.R	No literature was found
63	Supramarginal gyrus	SMG.L	(Grignon et al., 1998)
64	Supramarginal gyrus	SMG.R	(Grignon et al., 1998)
65	Angular gyrus	ANG.L	No literature was found
66	Angular gyrus	ANG.R	No literature was found
67	Precuneus	PCUN.L	(Karas et al., 2007)
68	Precuneus	PCUN.R	(Karas et al., 2007)
69	Paracentral lobule	PCL.L	(Kang et al., 2013a)
70	Paracentral lobule	PCL.R	(Kang et al., 2013a)
71	Caudate nucleus	CAU.L	(Möller et al., 2015)
72	Caudate nucleus	CAU.R	(Möller et al., 2015)
73	Lenticular nucleus, putamen	PUT.L	No literature was found
74	Lenticular nucleus, putamen	PUT.R	No literature was found
75	Lenticular nucleus, pallidum	PAL.L	No literature was found

76	Lenticular nucleus, pallidum	PAL.R	No literature was found
77	Thalamus	THA.L	(He et al., 2015)
78	Thalamus	THA.R	(He et al., 2015)
79	Heschl gyrus	HES.L	No literature was found
80	Heschl gyrus	HES.R	No literature was found
81	Superior temporal gyrus	STG.L	(Paakki et al., 2010)
82	Superior temporal gyrus	STG.R	(Paakki et al., 2010)
83	Temporal pole: superior temporal gyrus	TPOsup.L	No literature was found
84	Temporal pole: superior temporal gyrus	TPOsup.R	No literature was found
85	Middle temporal gyrus	MTG.L	(Aubry et al., 2015)
86	Middle temporal gyrus	MTG.R	(Aubry et al., 2015)
87	Temporal pole: middle temporal gyrus	TPOmid.L	(Sturm et al., 2013)
88	Temporal pole: middle temporal gyrus	TPOmid.R	No literature was found
89	Inferior temporal gyrus	ITG.L	(Scheff et al., 2011)
90	Inferior temporal gyrus	ITG.R	(Scheff et al., 2011)

SUPPLEMENTARY TABLE 2| Ranking results of different measures for AAL template (90 regions). The filling color of yellow indicates that the brain area has been verified to be related to AD.

NC_B	NC_C	NN_MC	NS_2hop	NC_D	GFS
78	3	37	42	3	40
37	40	40	3	9	3
40	9	38	40	22	37
29	37	78	22	40	42
68	78	77	49	15	22
42	22	15	16	42	78
3	42	42	58	37	15
58	15	68	15	59	9
1	89	67	7	78	68
77	58	73	45	89	38
7	59	71	23	16	77
59	68	22	2	58	58
38	16	48	8	21	16
89	38	31	10	1	29
84	29	29	50	14	89
60	77	3	1	29	59
8	1	72	9	53	1
22	84	5	13	79	7
16	7	21	59	7	49
43	21	6	17	8	84
15	60	89	14	10	21
9	14	82	62	27	8
82	53	27	5	60	27
79	27	9	27	38	23
53	49	58	6	68	43
30	79	30	60	84	67
39	8	43	51	5	71
51	10	84	43	77	2
2	23	16	53	49	5
80	71	49	67	2	60
88	43	47	70	23	10
23	2	28	37	80	14
14	5	32	71	43	48
54	30	1	68	51	53
49	48	88	28	17	79
10	51	23	4	30	30
27	67	25	69	88	51
24	80	33	21	13	88
21	88	2	63	48	6

12	13	81	19	71	80
70	39	19	11	39	17
55	17	4	79	25	13
48	25	10	41	24	39
17	82	39	89	67	28
45	47	14	77	6	47
65	24	24	85	28	24
44	6	51	81	47	25
67	28	46	48	82	45
5	55	59	57	55	73
36	46	7	61	4	55
19	4	86	75	46	82
47	61	55	47	54	19
18	54	41	87	26	70
66	26	60	65	44	46
62	44	8	24	45	61
71	45	17	55	61	44
25	70	79	83	73	4
28	73	26	84	70	62
35	65	13	88	12	54
46	12	53	44	19	50
13	19	75	25	41	65
61	62	80	30	86	12
69	41	61	80	81	26
6	86	45	73	62	81
86	81	54	12	72	41
64	32	50	29	32	86
26	50	90	35	50	32
63	75	69	32	65	75
50	57	44	46	83	69
11	72	57	38	33	57
52	83	65	39	57	66
75	36	70	64	75	35
41	35	63	26	66	72
73	85	62	90	36	85
81	66	36	66	35	83
32	33	52	86	85	36
85	64	66	31	31	64
87	69	64	33	18	11
57	11	85	54	11	63
20	18	12	78	64	31
76	31	76	20	69	33
4	52	87	72	52	18

83	63	18	52	63	52
90	90	83	36	90	90
34	20	35	76	76	87
33	76	56	56	56	76
72	56	20	18	20	20
56	87	11	82	34	56
31	34	74	34	87	34
74	74	34	74	74	74

SUPPLEMENTARY TABLE 3 | Comparison of the proportion of verified AD-related brain regions by different measures.

Rank	NC_B	NC_C	NN_MC	NS_2hop	NC_D	GFS
1	100.00%	100.00%	100.00%	100.00%	100.00%	100.00%
2	100.00%	100.00%	100.00%	100.00%	100.00%	100.00%
3	100.00%	100.00%	100.00%	100.00%	100.00%	100.00%
4	100.00%	100.00%	100.00%	100.00%	100.00%	100.00%
5	100.00%	100.00%	100.00%	100.00%	100.00%	100.00%
6	100.00%	100.00%	100.00%	100.00%	100.00%	100.00%
7	100.00%	100.00%	100.00%	100.00%	100.00%	100.00%
8	100.00%	100.00%	100.00%	100.00%	100.00%	100.00%
9	100.00%	100.00%	100.00%	100.00%	100.00%	100.00%
10	100.00%	100.00%	90.00%	90.00%	100.00%	100.00%
11	100.00%	100.00%	90.91%	90.91%	100.00%	100.00%
12	100.00%	100.00%	91.67%	83.33%	100.00%	100.00%
13	100.00%	100.00%	92.31%	84.62%	100.00%	100.00%
14	100.00%	100.00%	85.71%	85.71%	100.00%	100.00%
15	93.33%	100.00%	86.67%	86.67%	93.33%	100.00%
16	93.75%	100.00%	87.50%	87.50%	93.75%	100.00%
17	94.12%	100.00%	88.24%	88.24%	94.12%	100.00%
18	94.44%	94.44%	83.33%	83.33%	88.89%	100.00%
19	94.74%	94.74%	84.21%	84.21%	89.47%	100.00%
20	95.00%	95.00%	80.00%	80.00%	90.00%	95.00%
21	95.24%	95.24%	80.95%	76.19%	90.48%	95.24%
22	95.45%	90.91%	81.82%	72.73%	90.91%	95.45%
23	95.65%	91.30%	82.61%	69.57%	91.30%	95.65%
24	91.67%	91.67%	83.33%	70.83%	91.67%	95.83%
25	92.00%	92.00%	84.00%	68.00%	92.00%	96.00%
26	92.31%	88.46%	84.62%	69.23%	88.46%	96.15%
27	92.59%	88.89%	85.19%	70.37%	85.19%	96.30%
28	92.86%	89.29%	82.14%	71.43%	85.71%	92.86%
29	89.66%	89.66%	82.76%	72.41%	86.21%	89.66%
30	86.67%	90.00%	83.33%	73.33%	83.33%	90.00%
31	83.87%	90.32%	83.87%	74.19%	83.87%	90.32%
32	84.38%	87.50%	84.38%	75.00%	81.25%	87.50%
33	81.82%	84.85%	81.82%	75.76%	81.82%	87.88%
34	79.41%	85.29%	82.35%	76.47%	82.35%	88.24%
35	80.00%	85.71%	80.00%	77.14%	80.00%	85.71%
36	80.56%	86.11%	80.56%	75.00%	80.56%	86.11%
37	81.08%	86.49%	81.08%	75.68%	78.38%	86.49%
38	81.58%	84.21%	78.95%	76.32%	76.32%	84.21%
39	82.05%	82.05%	76.92%	76.92%	76.92%	82.05%
40	80.00%	80.00%	77.50%	75.00%	77.50%	80.00%

41	80.49%	80.49%	75.61%	73.17%	78.05%	78.05%
42	80.95%	78.57%	73.81%	71.43%	78.57%	76.19%
43	81.40%	79.07%	74.42%	72.09%	79.07%	76.74%
44	79.55%	79.55%	75.00%	72.73%	79.55%	77.27%
45	77.78%	80.00%	73.33%	73.33%	77.78%	77.78%
46	76.09%	80.43%	73.91%	73.91%	78.26%	78.26%
47	76.60%	78.72%	74.47%	74.47%	78.72%	78.72%
48	77.08%	79.17%	72.92%	75.00%	79.17%	77.08%
49	75.51%	79.59%	73.47%	75.51%	79.59%	75.51%
50	76.00%	78.00%	74.00%	74.00%	78.00%	76.00%
51	74.51%	76.47%	74.51%	72.55%	76.47%	76.47%
52	75.00%	75.00%	75.00%	73.08%	75.00%	75.00%
53	73.58%	73.58%	75.47%	73.58%	75.47%	75.47%
54	72.22%	74.07%	75.93%	72.22%	75.93%	74.07%
55	70.91%	74.55%	76.36%	72.73%	74.55%	72.73%
56	71.43%	73.21%	75.00%	73.21%	73.21%	73.21%
57	71.93%	73.68%	73.68%	71.93%	71.93%	71.93%
58	72.41%	72.41%	74.14%	70.69%	72.41%	70.69%
59	72.88%	71.19%	72.88%	69.49%	71.19%	69.49%
60	71.67%	70.00%	73.33%	70.00%	70.00%	70.00%
61	70.49%	68.85%	72.13%	70.49%	70.49%	68.85%
62	69.35%	67.74%	70.97%	70.97%	70.97%	67.74%
63	69.84%	68.25%	69.84%	69.84%	71.43%	68.25%
64	68.75%	68.75%	68.75%	68.75%	70.31%	68.75%
65	69.23%	69.23%	67.69%	67.69%	70.77%	69.23%
66	69.70%	68.18%	68.18%	68.18%	69.70%	69.70%
67	70.15%	68.66%	68.66%	68.66%	70.15%	68.66%
68	70.59%	67.65%	69.12%	67.65%	69.12%	67.65%
69	71.01%	68.12%	69.57%	66.67%	68.12%	68.12%
70	70.00%	68.57%	70.00%	67.14%	67.14%	68.57%
71	70.42%	67.61%	69.01%	67.61%	67.61%	67.61%
72	69.44%	68.06%	69.44%	68.06%	66.67%	68.06%
73	69.86%	68.49%	69.86%	68.49%	65.75%	68.49%
74	68.92%	68.92%	68.92%	68.92%	66.22%	68.92%
75	69.33%	68.00%	69.33%	68.00%	66.67%	68.00%
76	68.42%	67.11%	69.74%	68.42%	67.11%	68.42%
77	68.83%	67.53%	68.83%	67.53%	66.23%	68.83%
78	69.23%	67.95%	69.23%	66.67%	65.38%	67.95%
79	69.62%	67.09%	69.62%	65.82%	64.56%	68.35%
80	68.75%	66.25%	68.75%	66.25%	65.00%	67.50%
81	67.90%	65.43%	67.90%	65.43%	65.43%	66.67%
82	67.07%	65.85%	68.29%	65.85%	65.85%	65.85%
83	66.27%	66.27%	67.47%	66.27%	66.27%	66.27%

84	66.67%	66.67%	66.67%	66.67%	66.67%	66.67%
85	65.88%	65.88%	67.06%	65.88%	65.88%	67.06%
86	65.12%	65.12%	67.44%	66.28%	66.28%	66.28%
87	65.52%	65.52%	66.67%	65.52%	65.52%	65.52%
88	65.91%	65.91%	65.91%	65.91%	64.77%	65.91%
89	65.17%	65.17%	65.17%	65.17%	65.17%	65.17%
90	64.44%	64.44%	64.44%	64.44%	64.44%	64.44%

REFERENCES

Aubry, S., Shin, W., Crary, J. F., Lefort, R., Qureshi, Y. H., Lefebvre, C., et al. (2015). Assembly and interrogation of Alzheimer's disease genetic networks reveal novel regulators of progression. *PLoS ONE* 10, e0120352. doi: 10.1371/journal.pone.0120352

Beyer, N., Coulson, D. T. R., Heggarty, S., Ravid, R., Hellemans, J., Irvine, G. B., et al. (2012). Zinc transporter mRNA levels in Alzheimer's disease postmortem brain. *J Alzheimers Dis* 29, 863–873. doi: 10.3233/JAD-2012-112105

Eskildsen, S. F., Coupé, P., Fonov, V. S., Pruessner, J. C., and Collins, D. L. (2015). Structural imaging biomarkers of Alzheimer's disease: predicting disease progression. *Neurobiol Aging* 36 Suppl 1, S23-31. doi: 10.1016/j.neurobiolaging.2014.04.034

Foundas, A. L., Leonard, C. M., Mahoney, S. M., Agee, O. F., and Heilman, K. M. (1997). Atrophy of the hippocampus, parietal cortex, and insula in Alzheimer's disease: a volumetric magnetic resonance imaging study. *Neuropsychiatry Neuropsychol Behav Neurol* 10, 81–89.

Grignon, Y., Duyckaerts, C., Bennecib, M., and Hauw, J. J. (1998). Cytoarchitectonic alterations in the supramarginal gyrus of late onset Alzheimer's disease. *Acta Neuropathol* 95, 395–406. doi: 10.1007/s004010050816

Hallam, B., Chan, J., Gonzalez Costafreda, S., Bhome, R., and Huntley, J. (2020). What are the neural correlates of meta-cognition and anosognosia in Alzheimer's disease? A systematic review. *Neurobiol Aging* 94, 250–264. doi: 10.1016/j.neurobiolaging.2020.06.011

He, W., Liu, D., Radua, J., Li, G., Han, B., and Sun, Z. (2015). Meta-analytic comparison between PIB-PET and FDG-PET results in Alzheimer's disease and MCI. *Cell Biochem Biophys* 71, 17–26. doi: 10.1007/s12013-014-0138-7

Kang, K., Lee, H.-W., and Yoon, U. (2013a). P4-375: Idiopathic normal-pressure hydrocephalus, Alzheimer's disease and cortical thinning. *Alzheimer's & Dementia* 9, P872-P872. doi: 10.1016/j.jalz.2013.08.208

Kang, K., Yoon, U., Lee, J.-M., and Lee, H.-W. (2013b). Idiopathic normal-pressure hydrocephalus, cortical thinning, and the cerebrospinal fluid tap test. *Journal of the Neurological Sciences* 334, 55–62. doi: 10.1016/j.jns.2013.07.014

Karas, G., Scheltens, P., Rombouts, S., van Schijndel, R., Klein, M., Jones, B., et al. (2007). Precuneus atrophy in early-onset Alzheimer's disease: a morphometric structural MRI study. *Neuroradiology* 49, 967–976. doi: 10.1007/s00234-007-0269-2

Lehmann, M., Ghosh, P. M., Madison, C., Laforce, R., Corbetta-Rastelli, C., Weiner, M. W., et al. (2013). Diverging patterns of amyloid deposition and hypometabolism in clinical variants of probable Alzheimer's disease. *Brain* 136, 844–858. doi: 10.1093/brain/aws327

Li, J., Jin, D., Li, A., Liu, B., Song, C., Wang, P., et al. (2019). ASAF: altered spontaneous activity fingerprinting in Alzheimer’s disease based on multisite fMRI. *Science Bulletin* 64, 998–1010. doi: 10.1016/j.scib.2019.04.034

Liu, X., Chen, X., Zheng, W., Xia, M., Han, Y., Song, H., et al. (2018). Altered Functional Connectivity of Insular Subregions in Alzheimer's Disease. *Front Aging Neurosci* 10, 107. doi: 10.3389/fnagi.2018.00107

Liu, Y., Chen, Y., Liang, X., Li, D., Zheng, Y., Zhang, H., et al. (2020). Altered Resting-State Functional Connectivity of Multiple Networks and Disrupted Correlation With Executive Function in Major Depressive Disorder. *Front Neurol* 11, 272. doi: 10.3389/fneur.2020.00272

Ma, D., Fetahu, I. S., Wang, M., Fang, R., Li, J., Liu, H., et al. (2020). The fusiform gyrus exhibits an epigenetic signature for Alzheimer's disease. *Clin Epigenetics* 12, 129. doi: 10.1186/s13148-020-00916-3

Möller, C., Dieleman, N., van der Flier, W. M., Versteeg, A., Pijnenburg, Y., Scheltens, P., et al. (2015). More atrophy of deep gray matter structures in frontotemporal dementia compared to Alzheimer's disease. *J Alzheimers Dis* 44, 635–647. doi: 10.3233/JAD-141230

Mölsä, P. K., Säkö, E., Paljärvi, L., Rinne, J. O., and Rinne, U. K. (1987). Alzheimer's disease: neuropathological correlates of cognitive and motor disorders. *Acta Neurol Scand* 75, 376–384. doi: 10.1111/j.1600-0404.1987.tb05465.x

Paakki, J.-J., Rahko, J., Long, X., Moilanen, I., Tervonen, O., Nikkinen, J., et al. (2010). Alterations in regional homogeneity of resting-state brain activity in autism spectrum disorders. *Brain Res* 1321, 169–179. doi: 10.1016/j.brainres.2009.12.081

Qin, C., Liang, Y., Tan, X., Leng, X., Lin, H., Zeng, H., et al. (2019). Altered Whole-Brain Functional Topological Organization and Cognitive Function in Type 2 Diabetes Mellitus Patients. *Front Neurol* 10, 599. doi: 10.3389/fneur.2019.00599

Ren, S., Huang, Q., Jiang, D., Huang, L., Wang, Y., Guan, Y., et al. (2020). Brain amyloid accumulation and glucose hypometabolism in Chinese Alzheimer’s disease population. *Alzheimer's & Dementia* 16. doi: 10.1002/alz.043567

Reyes, P. F., Fagel, P., and Golden, G. (1986). THE OLFACTORY CORTEX IN ALZHEIMER’S DISEASE. *Journal of Neuropathology and Experimental Neurology* 45, 341. doi: 10.1097/00005072-198605000-00084

Scheff, S. W., Price, D. A., Ansari, M. A., Roberts, K. N., Schmitt, F. A., Ikonomovic, M. D., et al. (2015). Synaptic change in the posterior cingulate gyrus in the progression of Alzheimer's disease. *J Alzheimers Dis* 43, 1073–1090. doi: 10.3233/JAD-141518

Scheff, S. W., Price, D. A., Schmitt, F. A., Scheff, M. A., and Mufson, E. J. (2011). Synaptic loss in the inferior temporal gyrus in mild cognitive impairment and Alzheimer's disease. *J Alzheimers Dis* 24, 547–557. doi: 10.3233/JAD-2011-101782

Schultz, S. A., Larson, J., Oh, J., Koscik, R., Dowling, M. N., Gallagher, C. L., et al. (2015). Participation in cognitively-stimulating activities is associated with brain structure and cognitive function in preclinical Alzheimer's disease. *Brain Imaging Behav* 9, 729–736. doi:

10.1007/s11682-014-9329-5.
Shan, Y., Wang, J.-J., Wang, Z.-Q., Zhao, Z.-L., Zhang, M., Xu, J.-Y., et al. (2018). Neuronal Specificity of Acupuncture in Alzheimer's Disease and Mild Cognitive Impairment Patients: A Functional MRI Study. *Evid Based Complement Alternat Med* 2018, 7619197. doi: 10.1155/2018/7619197
Sturm, V. E., Yokoyama, J. S., Seeley, W. W., Kramer, J. H., Miller, B. L., and Rankin, K. P. (2013). Heightened emotional contagion in mild cognitive impairment and Alzheimer's disease is associated with temporal lobe degeneration. *Proc Natl Acad Sci U S A* 110, 9944–9949. doi: 10.1073/pnas.1301119110
Tsuchiya, K., and Kosaka, K. (1990). Neuropathological study of the amygdala in presenile Alzheimer's disease. *Journal of the Neurological Sciences* 100, 165–173. doi: 10.1016/0022-510X(90)90029-M
Vasconcelos, L. G., Jackowski, A. P., Oliveira, M. O., Ribeiro Flor, Y. M., Souza, A. A., Bueno, O. F., et al. (2014). The thickness of posterior cortical areas is related to executive dysfunction in Alzheimer's disease. *Clinics* 69, 28–37. doi: 10.6061/clinics/2014(01)05
Zhang, Y., Dong, Z., Phillips, P., Wang, S., Ji, G., Yang, J., et al. (2015). Detection of subjects and brain regions related to Alzheimer's disease using 3D MRI scans based on eigenbrain and machine learning. *Front Comput Neurosci* 9, 66. doi: 10.3389/fncom.2015.00066
